# Using Gaze Tracking as a Research Tool in the Deaf Health Literacy and Access to Health Information Project: Protocol for a Multisite Mixed Methods Study and Preliminary Results

**DOI:** 10.2196/26708

**Published:** 2021-09-07

**Authors:** Sara Champlin, Jessica Cuculick, Peter C Hauser, Kelley Wyse, Michael M McKee

**Affiliations:** 1 Mayborn School of Journalism The University of North Texas Denton, TX United States; 2 Department of Liberal Studies National Technical Institute for the Deaf Rochester Institute of Technology Rochester, NY United States; 3 Research Center on Culture and Language National Technical Institute for the Deaf Rochester Institute of Technology Rochester, NY United States; 4 Family Medicine Department The University of Michigan Ann Arbor, MI United States

**Keywords:** gaze tracking, deaf, disabilities, accessibility, online health information, health information seeking, mobile phone

## Abstract

**Background:**

Previous studies have identified the internet as a major source of health information. Reliable and accessible sources of web-based health information are critical for cultivating patient-centered care. However, the accessibility and use of web-based health information remains largely unknown for deaf individuals. We used gaze-tracking technology to understand the navigation and use of web-based health information by deaf adults who communicate with sign language and by hearing adults.

**Objective:**

This paper discusses our protocol for implementing gaze-tracking technology in a study that included both deaf and hearing participants. We report the preliminary results and lessons learned from the implementation of the protocol.

**Methods:**

We conducted gaze-tracking sessions with 450 deaf signers and 450 hearing participants as a part of a larger, multisite mixed methods research study. Then, we conducted qualitative elicitation interviews with a subsample of 21 deaf and 13 hearing participants, who engaged in a search task and reviewed their gaze recordings. To our knowledge, no study has implemented a similar research protocol to better understand the experiences of deaf adults. As such, we also examined research staff notes and observations from team meetings regarding the conduct of gaze-tracking data to delineate lessons learned and best practices for research protocols in this area.

**Results:**

Findings from the implementation of this study protocol highlight the use of gaze technology with deaf participants. We developed additional protocol steps to minimize gaze disruption from either lipreading or communicating in sign language. For example, research assistants were often unable to maintain eye contact with participants while signing because of the need to simultaneously point at the computer monitor to provide instructions related to gaze study components, such as the calibration process. In addition to developing ways to effectively provide instructions in American Sign Language, a practice exercise was included in the gaze tracker session to familiarize participants with the computer and technology. The use of the playback feature permitted a deeper dialogue between researchers and participants, which we found vital for understanding the experiences of deaf participants.

**Conclusions:**

On the basis of our experience using the study protocol through a large research project, incorporating gaze-tracking technology offers beneficial avenues for better understanding how individuals interact with health information. Gaze tracking can determine the type and placement of visual content that attracts attention from the viewers of diverse backgrounds, including deaf individuals. The lessons learned through this study will help future researchers in determining ideal study designs, such as suitable protocols and participant characteristics (eg, deaf signers), while including gaze trackers in their projects. This approach explored how different ways of presenting health information can affect or enable visual learners to engage and use health information effectively.

**International Registered Report Identifier (IRRID):**

RR1-10.2196/26708

## Introduction

### Background

Between 40% and 60% of adults in the United States report regularly engaging in an internet search for information about a health topic [1,2]. Recent data from Google Health suggest that 7% of daily searches on the engine are for health topics [3]. The internet remains the most accessed source of health information, beyond health care professionals, traditional media, friends, and family [1]. This is to be expected, as changes within the health care system, including patient-centered care, require that individuals become more actively involved in their own health, including pursuing and managing information [4,5]. Adults are expected to take charge of their health decisions. Similarly, the development of health information technology (HIT), such as electronic health care records, patient portals, health apps for smartphones, and wearable technology, adds to the ways adults can directly monitor their health and participate as engaged communicators and consumers of health care.

Although these advancements can improve health outcomes for some, research suggests that they exacerbate health disparities for many, including those with disabilities [6-11]. The COVID-19 pandemic again exposed these inequities as it “ushered in a new era of telehealth” [11]. This has resulted in an increased need for medical visits provided through virtual options [11,12] and the dissemination of information through web-based sources [13]. During the rapidly evolving health crisis, those with disabilities were left behind, with critical health information often not made available in other languages or access options [14,15]. For example, the White House did not include American Sign Language (ASL) interpreters at their COVID-19 press briefings [14]. Telehealth options remained mostly inaccessible to those with disabilities or without reliable internet access [11]. Hence, a health *infodemic* was born, in which health information available on the internet often contained incorrect or outdated recommendations [13,16]. These injustices are likely to persist without more research and consideration of how web-based health information and HIT are developed [11].

Goldberg et al [17] argue, “missing from the national efforts toward pervasive ability for HIT for adults, families, doctors, and health care facilities is a programmatic and policy-based effort to ensure that people with disabilities (PWD) are able to participate equally in all the opportunities that new Health 2.0 networks and tools have to offer...”. Indeed, health information websites have historically been found to be inconsistent in the extent to which accessibility requirements were met, which affects who can use this content and how they use it [18]. Unfortunately, this is a vicious cycle as a majority of the existing research on web-based health information seeking has been conducted with adults without disabilities, which severely limits the understanding of how people with disabilities access this content and opportunities for improvement. More work is needed to understand people with disabilities’ access to and experience with web-based information.

### Deaf Adults and Health Information on the Web

The national estimates suggest that nearly one million Americans are deaf signers [19]. The internet is a primary source of health information for this audience and played an important role for deaf adults during the pandemic [20]. Greater attention in improving the accessibility and user experience of health information on the internet is especially imperative for deaf adults, for whom there is relatively little available research [6,21]. Specific calls have been made to software developers and designers to improve the accessibility of web-based health information and technologies [11]. Deaf individuals encounter significant communication barriers, resulting in lower patient-provider satisfaction, adherence, inappropriate health care utilization, and decreased engagement in health-related decision-making [21,22]. As a result, deaf individuals are almost seven times more likely to have less than adequate health literacy. To help with the low availability of health knowledge (defined as limitation in one’s factual knowledge base as compared with the general population) due to inaccessible information and loss of incidental learning opportunities [23], deaf adults must then turn to community peer exchanges, print media, family and friends, and the internet to obtain health information [24].

One study demonstrated that 79% of deaf individuals use the internet daily and are almost three times more likely to search the internet for health information than their hearing peers [25]. However, deaf adults may find navigating the internet to be a challenging task [26]. Some deaf individuals display competency in navigating visual targets but struggle with categorical decision-making (often based on semantics rather than visual cues) to make a more refined search, such as navigating through a series of pages to reach information that exists deep within a website [27]. Most web-based health information requires more categorical decision-making because of the level of semantics and textual-based graphics.

Although it is known that deaf adults access the internet at higher rates to retrieve health information and guidance [25], the navigation, barriers, and specifics of these searches are underresearched. Exploring these actions can shed light on the objective ways this community engages in web-based health information seeking. Karras and Rintamaki [6] describe the potential for the internet to serve as a “double-edged sword in that it provides boons and challenges for deaf people.” It is important to devote greater research attention on how deaf adults access and use health information on the internet [6], including the steps taken, assessments of content credibility, and accessibility of this information for various audiences. Specific consideration should be devoted to deaf adults so that health disparities among these populations can be reduced.

### Objectives

The purpose of this paper is to introduce a research methodology, the use of gaze tracking and visualization, to gain new insights into this specific population. Gaze-tracking technology affords numerous research opportunities and can be implemented with participants of all ages and many different backgrounds but has not been widely implemented in people with different types of disabilities. For health communication researchers, gaze-tracking data can contribute to naturalistic, objective findings about information seeking and engagement that cannot be assessed in other ways. Although there are numerous benefits to this useful tool, there are a number of best practices and recommendations that researchers should consider before choosing to embark on this endeavor. We outline the preliminary results observed while implementing this protocol in the context of a larger study.

Researchers interested in the extent and ways in which deaf adults engage with health information can benefit from studying these adults’ eye behaviors. A great deal of health information is visual in nature. This includes pamphlets, web-based content, videos, infographics, recommendations and guidelines, health procedure prep instructions, handwritten notes, drawings, graphs, and countless other forms. The exploration of how deaf adults direct their visual attention is essential for the work of health communication scholars, as it can help us understand what draws adults to specific information and thus guide best practices for designing health content that is engaging and accessible for adults of different backgrounds. In addition, design quality and visual design assessments have been shown to affect the perceived credibility of health content as well as influence attitudes and comprehension [28,29].

In the remainder of this paper, we provide an overview of gaze-tracking technology and how we implemented this protocol in a large-scale, multisite mixed methods research study involving both deaf and hearing participants. The goal of the gaze-tracking component was to better understand deaf participants’ search and information scanning behaviors in response to examples of internet health information websites. Finally, recommendations for using this methodology are outlined, based on the preliminary results.

## Methods

### Overview of Gaze Tracking

Humans are not physically equipped to attend to all possible visual stimuli; thus, we engage in selective attention to conserve cognitive resources. When adults perform searches, scan, read, or extract details about health topics, they are engaged in selective visual attention—deciding where to spend time looking and processing information. Studying eye movements and visual attention sheds light on the “what” and “where” conditions under which stimuli gain attention [30]. Humans move objects of interest into a visible field so that they may examine them further. This results in a gaze path that reflects different points of visual attention exhibited by a viewer [30].

A gaze-tracking system is a type of technology that measures a participant’s eye movements in response to a visual stimulus. As a methodology, gaze tracking offers quantitative data regarding how long a participant spends looking at a given stimulus as well as the specific aspects that gain the most attention. Previous reports suggest that pairing patient-reported survey data with gaze tracking and qualitative responses, or “methodological triangulation,” can offer deeper interpretations of this behavior [31].

Gaze tracking is one of only a limited set of objective assessments that capture attention in a naturalistic setting and is perhaps the leader in affordability, practicality of use, and the ease of data interpretation [31,32]. Visual attention can also be captured using brain imaging techniques such as functional magnetic resonance imaging [33,34], but this methodology warrants steeper learning curves and higher costs for the user.

Gaze tracking is a true-to-life research methodology that can capture the eye movements and patterns in everyday life with minimal intrusion. Understanding real-time, naturalistic searching and reading is essential for building content for adults—this starts with understanding what they are already doing, rather than guessing and building ineffective interventions.

### Study Design and Participants

A large, multisite, explanatory sequential mixed methods study was conducted with deaf and hearing participants at three locations [26]. The participants self-identified their hearing status and the languages they use. All study materials were provided to deaf participants in ASL. As such, we recruited only deaf signers.

The overall study aimed to address the factors influencing health literacy in each of these two groups. The goal of the gaze-tracking component of the study was to assess and understand participants’ search and information scanning behaviors in response to examples of health information websites. Given the limited research in this area, we aimed to outline these factors using quantitative gaze tracking and survey assessments in the first phase of the study and then explore them in greater depth using qualitative methods in the second phase of the study. Thus, the mixed methods design allowed us to examine not just *what* participants looked at on the internet and the factors that influenced this search (phase 1) but also *how* they found this information and *why* they pursued specific content (phase 2). This was designed based on previous work by a mixed methodologist on the team [35-38] and was an appropriate fit for the goals of the project. Through this National Institutes of Health–funded grant, we were able to explore the capabilities and limitations of gaze-tracking research in the context of how deaf and hearing adults find, use, and understand health information on the internet.

In the first phase of the study, a series of quantitative cross-sectional survey items and tasks were implemented among 450 deaf and 450 hearing participants to identify the predictors and moderators of health literacy between these groups. We integrated a gaze-tracking component into this phase of a larger study. In this experimental aspect of the study, participants viewed the US Centers for Disease Control and Prevention (CDC) webpages of four health conditions and answered questions about the page. The development of these stimuli and procedures are described in greater detail in the *Developing Study Stimuli* section. Participants viewed the CDC webpages on a laptop to simulate what they might do if they were looking for health information on the internet in their everyday lives.

Through this first phase of the study, we assessed the predictors for adequate health literacy and the ability to use web-based health information and found several key variables, such as age, race and ethnicity, language fluency, and reading literacy. A diverse set of participants with these backgrounds stratified by health literacy adequacy were invited back for the second phase, which incorporated a task performance and an elicitation interview.

In the second phase, this sample of participants, selected using the quantitative data obtained in the first phase, was asked to perform a search task and review their gaze-tracking results through the technology’s playback feature and engage in elicitation interviews in response to these results. This qualitative component was guided by previous research and study design that implemented video elicitation interviews [36,37]. This second phase provided greater clarity on how and why deaf individuals access and understand web-based health information, as it explored both the “complex cognitive or decision-making processes and participants’ reactions to or assessments of their own actions” while accessing web-based health information [36].

### Equipment Used

We implemented the Tobii Pro X3 system for this project. This gaze-tracking system is easy to use and highly mobile given its small size (approximately 7 inches in length). The multisite nature of this project enabled the research team to store, pack, and move the system with ease. To set up the system for data collection, the bar-shaped tracker was clipped to a computer monitor using a strong magnet. As such, the tracker could be easily reattached to other screens and computers with the addition of a magnet to each new screen. The X3 is designed to capture gaze data on laptop and computer monitor screens [39]. We selected the 120 Hz version as we were interested in examining participants’ scanning of several visual areas of interest (AOIs) and the nuanced attention on specific words on the screen. This model has since been discontinued by Tobii, but a similar model, the Tobii Pro Fusion, is comparable.

### Developing Study Stimuli

The Tobii X3 system can accommodate many forms of media, including real-time website browsing, static images (such as nutrition or medicine labels, consent forms, decision aids, and messages), videos, surveys, and other components. We implemented a *web element* and *questionnaire* elements for this study. Furthermore, we created multiple sessions or *conditions* through which participants viewed content for comparison.

**Figure 1:**
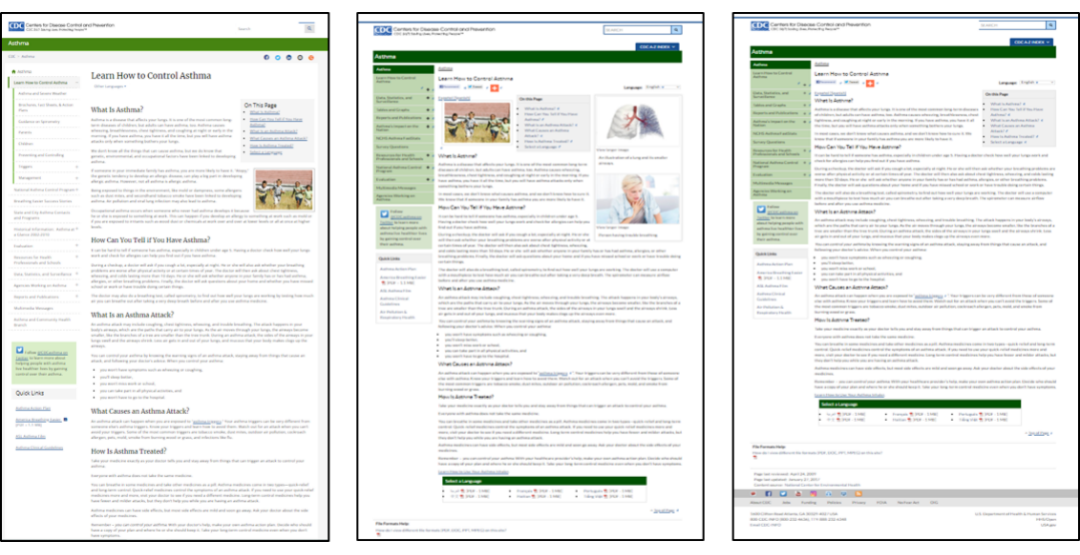
Sample stimuli from left to right: (1) actual Centers for Disease Control and Prevention webpage, (2) experimental study stimuli with pictures, (3) experimental study stimuli without pictures.

One aim of the study was to explore the types of content on a health website that would gain attention from deaf and hearing participants from varied personal backgrounds. We found that using a *live* webpage (directly linked to a CDC webpage) would not suffice, as content could be changed or updated by the page owner on the site at any time. If the page owners changed the content during the span of data collection, this would introduce unwanted variability in our visual stimuli. Furthermore, using *live* content on the internet would lend authenticity and generalizability to the study; however, it would not allow us to manipulate the conditions. We included four health conditions, selected through pilot testing. Two were commonly known topics (asthma and sinusitis) and two were lesser known (Sjogren syndrome and staphylococcal food poisoning). We were also interested in how deaf and hearing adults would peruse and evaluate visual content. Each health condition also featured a version of the page with and without pictures (Figure 1).

We created static website images or *screenshots* for use within the study. We created PDF pages that mimicked the real content seen on the CDC website but were still able to manipulate the content as needed. Following this, we created links to each page on our lab server. Creating these links, and having the images available on the internet, allowed the use of the web element within the Tobii Studio software, which recorded the participant’s gaze pattern as they scrolled down a page, just as they would with a live website. Participants viewed the page content and answered questions based on what they read.

### Measures

**Figure 2:**
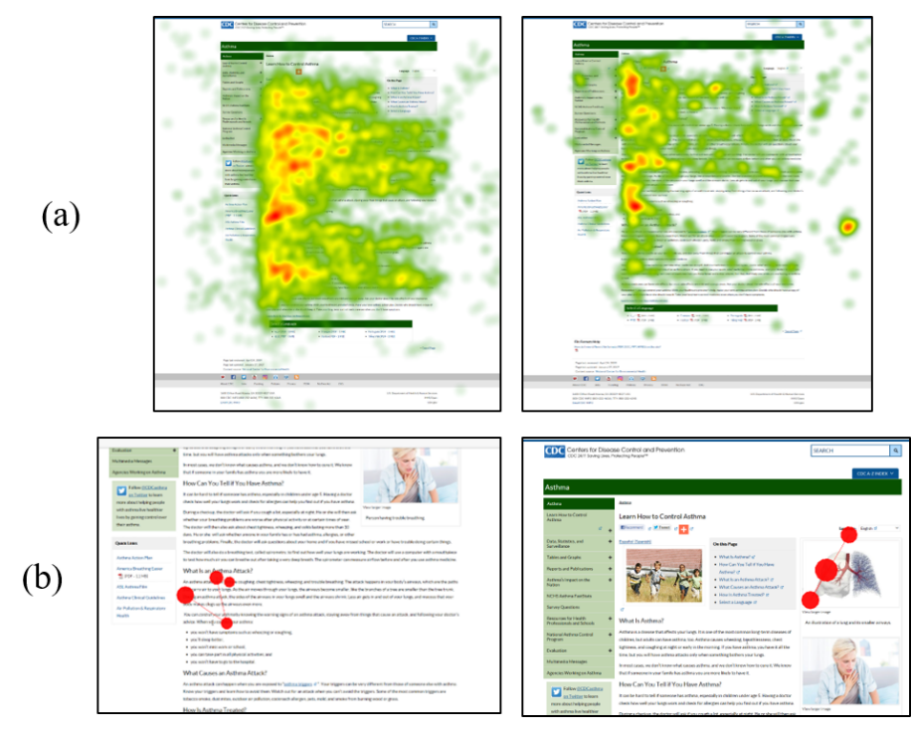
Examples of heat maps and gaze diagrams collected in this study: (a) Example heat map: group of low health literacy hearing participants (left), group of low health literacy deaf participants (right)—the gradient of colors (green to red) indicate the density of visual attention (low to high, respectively); (b) Example gaze plot of asthma survey component.

To capture attention, gaze trackers record a participant’s eye movements using built-in sensors and cameras. In many models, near-infrared light is projected from the gaze tracker onto a participant’s pupils [40]. The length of time a participant spends on a given stimulus (a variable referred to as *fixation duration*, reflected in seconds), the number of times a participant returns to the stimulus (*fixation count*, reflected in counts or hits), and the gaze path a participant performs while scanning the visual information are calculated in response to the ways in which the near-infrared light reflects off the pupil. These three variables (fixation duration, fixation count, and eye gaze paths) are common measures exported from a gaze-tracking system and were used in this study to reflect attention given to the various areas of the webpage. The first two variables capture the quantitative assessments of visual attention, whereas the gaze paths are presented in high-quality visualization images, such as heat maps and gaze diagrams (Figure 2), which allow for other forms of data analysis.

**Figure 3:**
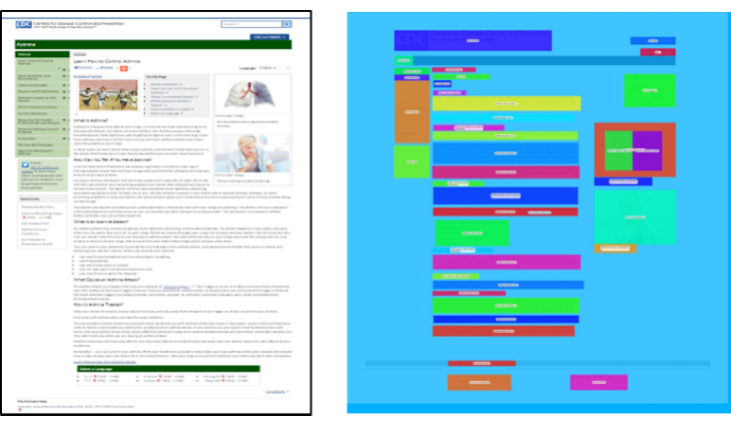
Example Area of Interest tool.

Moreover, we used the Tobii AOI tool to create visual *areas* by drawing boxes or shapes around specific visual content of focus (Figure 3). In our study, we were interested in how much visual attention was garnered by pictures included on a given website page. Using the AOI tool, we drew a box around the picture to create a visual area. The Tobii Studio software provides the aforementioned information (eg, fixation duration) for the AOI, in this case, a picture. AOIs can be created for any visual element on a page, such as headlines, click buttons, captions, and infographics.

**Figure 4:**
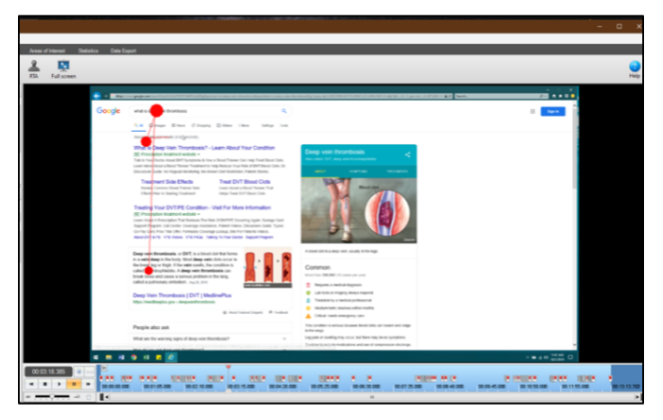
Example video replay. The participant searches for information about deep vein thrombosis.

For the qualitative elicitation interviews in phase 2 of the study, we implemented the playback video tool to review and discuss gaze-tracking recordings with the participant (Figure 4). After a gaze-tracking session, Tobii provides an opportunity to immediately view a recording of the participants’ gaze as they scan various stimuli. After completing the first phase of the study, we invited several participants back to peruse additional health information in a separate free search task (in which the participant was provided with a vignette and asked to find information on their own about the health topic, using any web-based searching methods and terms) and discuss how they made decisions to search and look at specific information.

### Recruitment and Screening

The recruitment and screening guidelines were essential components of this study. Importantly, we asked participants about their vision. During recruitment, we asked if participants had any visual impairments. If so, they were then asked whether they were able to see a computer screen easily. Participants indicated whether they wore eyeglasses, contacts, no glasses, had vision limitations, or were color blind. To avoid data collection or scheduling issues when participants forgot to bring their glasses, our research team was also equipped with a tray providing multiple reading glasses ranging from a +1.25 to +2.0 power in +0.25 power increments. Tobii [41] advises that glasses-wearing participants clean their eyewear before the study. As a result, glass cleaners and cloths were offered during the study session.

### Session Setup

#### Overview

In phase 1, the gaze-tracking component of this project was included in a battery of other tests included in the larger study. When it was their turn to complete the gaze-tracking task, the participant was asked to sit down in front of the laptop. Specifically, we selected a chair for participants that does not swivel and was both easy and comfortable to sit in, as participants may fidget or move in the chair, which can disrupt the collection of gaze data. The research assistant then performed the following phases.

#### Calibration

First, a research assistant performed a brief calibration process using the gaze-tracking software. Achieving an accurate calibration is important for data analysis, as this process will connect a participant’s gaze path with the corresponding content appearing on the screen. The calibration process involved watching a dot scan across the screen to different corners of the computer screen. Following this, the participant observed the visual stimuli on the screen as they would normally. As many of the participants had not participated in a gaze-tracking study before, it was essential that detailed explanations and instructions were given before beginning a session.

Research assistants provided instructions and explanations for what to expect regarding the calibration process. Researchers explained the importance of sitting in one spot for the study and remaining on the angle of the monitor (to the best of their ability) due to the tracking devices needed to retrieve the data. In addition, assistants discussed resisting the urge to break visual connections with the computer monitor, as this could lose the gaze data.

#### Acquainting Participants With Gaze Tracking

During our pilot phase, we found that the calibration and initial steps in the gaze-tracking session were cumbersome for deaf participants because they had to look back and forth between the monitor and the research assistant to receive instructions in ASL. We were concerned that participants would still be getting a feel for the study and its procedures well into the first health topic test session. As such, we added a practice health topic that mirrored the format of the other topics, but it was not used for data collection purposes. In this case, we used a screenshot of the CDC’s website about influenza (flu). In this practice session, participants read questions, viewed the page, and answered questions. This practice session helped put participants’ minds at ease as they got a feel of the study procedures. Participants could ask questions if anything was unclear. The intention of this test was to get participants who liked to make eye contact or lacked computer skills to become well oriented with the gaze-tracking session. We also explained how gaze trackers work and asked them to focus on the computer screen until their task was completed. This helped in reducing the proportion of gaze tracker failures due to breaks in gazes.

Once participants completed the practice session and felt comfortable with the gaze tracker, we asked them to proceed with the four CDC health condition webpages. Participants were randomized to view these four conditions with or without relevant pictures or graphics included alongside the text. Each participant saw one of four versions of the study, each of which presented the health topics in a different order. Participants were asked questions about each of the illnesses, then viewed a website screenshot of the corresponding CDC page, and subsequently asked to again answer the questions they saw before viewing the web content.

### Qualitative Elicitation Interviews

To gain a more in-depth understanding of the typical search and navigational abilities of our participants, a subsample of deaf and hearing participants with different levels of health literacy and other quantitative variables observed in phase 1 were invited back to participate in a second gaze-tracking session. Four brief clinical vignettes (ie, pneumonia, deep vein thrombosis, migraines, and appendicitis) with multiple-choice answers were provided to all participants as a way to prompt web-based searches. These vignette topics were selected through pilot testing and were chosen to avoid recall bias. The recording of the participants’ web-based activities was then reviewed together with the research assistant and used as a part of an elicitation interview to elucidate how and why deaf and hearing individuals access and understand different types of health information. The captured data from the gaze tracker allowed the interviewer to ask more detailed questions on how and why such an action was chosen and the participant’s thought process related to the web-based information. Similar to what was done in phase one, an influenza topic was used as a practice exercise to help participants become familiarized with the gaze tracker.

## Results

### Overview

Gaze-tracking technology provides an objective assessment of the visual content that draws attention from viewers of diverse backgrounds. We found that this type of data collection is especially useful while determining the barriers and challenges deaf adults have with health information on the internet and how it is presented. The gaze tracker recordings and their ability to play back or tag certain time points are useful for conducting elicitation or cognitive interviews or usability testing. It is important to note that we observed a learning curve while using this technology with deaf participants, namely, the importance of explaining to participants what they could expect during the session and what they were being asked to do. We also learned about the limitations gaze-tracking systems and projects have with large numbers of participants, including large file sizes. We will now describe preliminary findings related to our research protocol.

### Recruitment and Screening

Overall, we had high rates of participants who wore glasses. Having this information before the start of the study—and reminding participants that they will be reading information on a computer screen—is an important point for those who may use reading glasses. This will also help researchers understand what to expect before the participant arrives for the session. The Tobii gaze-tracking system has a “unique tolerance for eyewear” as compared with other systems and thus may be more conducive for use among participants with vision-related disabilities. Tobii [41] advises that glasses-wearing participants clean their eyewear before the study. Despite this, our research teams had difficulty in attaining successful calibration and gaze-tracking sessions with some glasses-wearing participants, particularly those who wore bifocals or progressives. For those with difficult calibration sessions due to their bifocal or progressive glasses, we encouraged participants to choose from our reading glasses of varying strengths to minimize this issue.

On the basis of our experience with this project, we would also include additional screening questions in future gaze-tracking projects, such as whether the participant can use a mouse with the computer and conduct a visual acuity and field screen to measure their visual abilities.

### Session Setup

#### Calibration

This was difficult for deaf participants who were simultaneously watching the research assistant explain the process using ASL. The research assistant would, at times, need to break eye contact with the participant. This was difficult to manage, as the research assistants would ideally be able to maintain eye contact with participants to facilitate the provision of instruction. While working with deaf participants, the researchers would point at the calibration dot and provide time for the participant to see where they were pointing and then pause for them to look at the signer. Through our experience with calibration, it would also be helpful to change the calibration dot to another color (eg, blue or purple), which is typically red by default and not easily detected, especially by those who have red-green colorblindness. This is a possibility in the Tobii Studio software program by navigating to *Calibration* tab under *Global Settings*. Finally, through our experience, it can be difficult to obtain gaze data with participants who do not read content straight on but prefer to read at an angle.

It is also important to note that the research team experienced some challenges regarding the distance from the participants’ eyes to the gaze tracker. There were instances in which researchers struggled with the seating angle of the participant to ensure that the visual distance was appropriate before testing. This was especially common among participants who were exceptionally short or tall. It can be helpful to have a practice text document open on the desktop of the computer to give participants an example of the text size included in the study materials. The participant may want to wear reading glasses or adjust how close they are to the screen. These adjustments should be made before starting the study session.

#### Session Duration and Complexity

Our project asked participants to complete a heavy amount of reading if they chose to read the entirety of the articles presented, which could be tiring for participants. For example, the page on asthma contained 725 words. In the case of our study, we wanted the gaze-tracking content to mimic a true, live webpage and thus chose to mirror our content, including its length, to that provided by the CDC. Likewise, the time of day can potentially play a role in the willingness or capability of a participant to engage with more content. Controlling for the time of day in which study sessions are scheduled (or counterbalancing through random assignment) can contribute to more accurate reflections of attention devoted to the study content.

During the qualitative elicitation interviews, upon completion of the clinical vignettes, participants were encouraged to take a short break of approximately 15 minutes. This break period allowed the research assistant to review the Tobii recording of the activity and video tag key times to use as prompts for the elicitation interviews. This step complemented the assistant’s field observation notes in preparation for the 1:1 elicitation interview with the participant. When the participant returned from the short break, the assistant and participant reviewed their Tobii recording together. The recording would then be used to learn about the participant’s thought processes during search queries, viewing patterns, and selection of websites.

However, because of participants’ need for periodic breaks in gaze connections to read the clinical vignette-based questions, several options were explored to minimize this. Once the Tobii project is started, it does not allow for switching back and forth to different media displays (eg, Internet Explorer to Word document listing the questions). This forced us to print out the questions. Clipping them up next to the computer versus laying in front of the computer did not appear to make much difference in terms of visual breaks and loss of gaze tracking. Our piloting phase revealed that short intermittent visual breaks are permissible with Tobii gaze trackers as long as the breaks are not lengthy. However, actual testing, especially among those with lower literacy levels, required longer visual breaks than expected. These visual breaks would accumulate over a period of 10-15 minutes and often would eventually result in a loss of gaze-tracking abilities for the remainder of the test. For those with a loss of gaze tracking, other elements were captured. The data included the video footage of the subject (including eye movements and facial expression), websites visited, duration of page visited, number of clicks, and cursor activity. The use of clinical vignettes to encourage typical web-based searches resulted in rich data points that included participants’ query formulations, navigation patterns, cursor activity, total search times, and number and nature of websites accessed, which can help explain the different abilities of deaf and hearing participants.

### Data Storage

Another unique aspect of this study was the use of multiple data collection sites, which has not been well described in previous research. We learned many lessons about using gaze-tracking systems across multiple data collection sites. Using multiple systems on the same project can offer tremendous benefits in terms of generalizability, but this practice also comes with considerable challenges. While creating a project, the data and sessions will be stored and can be analyzed locally, only on the laptop or computer using the Tobii Studio software. Although a session can be created on this machine and transferred to another one, it is not possible to merge these files unless one large project is created at the outset (rather than two identical but unlinked projects on separate machines). This created limitations for our study. We advise researchers working in this area to create one large project on a *master* computer and then share with computers at other data collection sites, rather than creating a *new* project on each of the individual computers. This will streamline the ability to compile data from each site.

Although an inherent benefit of gaze-tracking investigations is the real-time, second-by-second data collection, this makes for sizable individual data files. Specifically, the gaze-tracking files attained in this study ranged from 14.9 MB to 68.6 MB, with an average file size of 40.0 MB. For the elicitation interviews that allowed participants to perform real-life web-based searches, the files were as large as 17 GB. We were specifically challenged by the data storage and sharing capacities for our large-scale study. Limited storage space on a computer can cause the Tobii system to crash while data collection is in progress. This is problematic as restarting a data collection session disrupts and compromises the benefits of naturalistic observations offered through this research methodology.

Training to use the machine is another consideration for researchers. However, the software was not intuitive. Some of our researchers had an assistant familiar with the software to help with software navigation. Having an additional person in the room may compromise the naturalistic process for participants while they navigate health information on the internet.

## Discussion

### Principal Findings

The purpose of this study was to highlight the benefits and complexities of using a gaze tracker system to assess how deaf adults access health information and provide preliminary results in response to the implementation of this protocol. We learned that deaf signers may find aspects of the gaze-tracking session, such as calibration, challenging; however, these can be addressed in future studies through the use of this protocol paper. Gaze tracking is an affordable and effective way to understand how adults search and spend time processing patient-facing health information, such as that available on the internet. Previous research has articulated the ways in which gaze tracking can be successfully implemented in a research setting. These capacities were also observed in this study. However, our research team also experienced clear limitations with the system, notably with large sample sizes and data collection across research sites. Lessons learned also included considerations for extensive training for research assistants, as the software and procedures are not self-explanatory.

### Conclusions

The experiences learned through this study will help future researchers determine ideal study designs, such as suitable protocols and participant characteristics (eg, deaf signers), when including gaze trackers in their projects. The procedures we found most effective in working with these populations were discussed, and suggestions for future research were proposed.

## Abbreviations

AOI: area of interest
ASL: American Sign Language
CDC: US Centers for Disease Control and Prevention
HIT: health information technology
